# Functional Differentiation Reconfiguration in the Midgut of *Nezara viridula* (Hemiptera: Pentatomidae) Based on Transcriptomics: Multilayer Enrichment Analysis and Topological Network Interpretation

**DOI:** 10.3390/insects16060634

**Published:** 2025-06-16

**Authors:** Dongyue Yu, Jingyu Liang, Wenjun Bu

**Affiliations:** Institute of Entomology, College of Life Sciences, Nankai University, Tianjin 300071, China; yudongyue@mail.nankai.edu.cn (D.Y.); jljy47ky@163.com (J.L.)

**Keywords:** functional specialization, midgut regions, KEGG pathway enrichment, gene regulatory networks, pest control targets, Nezara viridula

## Abstract

The present investigation systematically elucidates the distinct functional specialization within the M1–M3 midgut sections of the significant agricultural pest, *Nezara viridula*. Employing an integrated transcriptomic analysis, three pivotal discoveries were achieved: (1) each midgut segment possesses unique gene expression signatures; (2) metabolic and signal transduction pathways exhibit coordinated regulatory patterns; and (3) parallel expression changes occur between neuroreceptor (e.g., *TACR*/*HTR*) and metabolic enzyme (e.g., *GLA*/*NAGA*) genes within identical midgut segments. These data reveal that the M1 region is primarily enriched in metabolic processes and neural signaling; the M2 region emphasizes cellular junctions and immune responses, while the M3 region is mainly responsible for cellular senescence and renewal. These discoveries advance the understanding of feeding adaptation mechanisms in Hemipteran insects and propose a “metabolism–defense–regeneration” functional model for the midgut. The established multi-level analytical framework provides a robust methodology for subsequent dissection of complex biological systems, identification of key molecular targets for functional validation, and for the development of novel pest control strategies.

## Simple Summary

Employing a systematic bioinformatics approach, this investigation elucidates the functional specialization mechanism formed in the midgut of *Nezara viridula* during adaptation to a maize-based diet, revealing a “metabolism–defense–regeneration” regulatory system with fine-grained functional compartmentalization. The research demonstrates significant functional differentiation among distinct midgut regions (M1, M2, M3): M1 primarily governs nutrient metabolism and detoxification and M2 emphasizes cell communication and immune defense, while M3 regulates cell renewal. Particularly noteworthy are the parallel enrichment patterns of neuro-metabolic pathways and the discovery of differentially expressed genes (such as *TACR*, *HTR*, *GLA*, *NAGA*), which provide novel insights into understanding insect dietary evolution, while the identified key genes offer potential targets for pest control. These achievements not only deepen the comprehension of insect digestive physiology but also establish a theoretical foundation for developing green management strategies against agricultural pests.

## 1. Introduction

The insect digestive system acts as a crucial interface connecting the external environment and internal physiological processes, playing central roles in nutrient acquisition, detoxification metabolism, and immune defense [1]. Specifically, the gastrointestinal tract, as the primary functional region of the insect digestive system, is responsible not only for food digestion and nutrient absorption but also serves as the defense line against external toxins and pathogens [2]. Recent advances in omics technologies have significantly enhanced the understanding of molecular mechanisms underlying insect midgut functions. However, systematic studies focusing on the functional differentiation among different midgut segments and their molecular foundations remain limited [3,4].

Notably, existing research has predominantly focused on model insects such as *Drosophila melanogaster* and *Bombyx mori* [5,6], leading to a relative lack of understanding regarding other agricultural pests. *Nezara viridula*, a significant agricultural pest with a broad host range and the cause of substantial economic losses [7], warrants dedicated investigation. Similar to many hemipteran insects, the midgut of *Nezara viridula* is morphologically categorized into four distinct sections: M1, M2, M3, and M4 [8]. This intricate structural division suggests considerable functional specialization among the different midgut regions. For instance, the M1 segment exhibits a distinct transcriptional profile and plays a crucial role in food digestion and detoxification, with studies showing enriched expression of proteolytic enzymes and xenobiotic metabolism enzymes in this anterior region. The M2 and M3 segments are also extensively implicated in digestion and detoxification processes. In contrast, the M4 segment is a highly specialized region for housing and maintaining symbiotic bacterial communities. These symbiotic bacteria establish a symbiotic relationship with the host, supporting normal host growth and survival by participating in nutrient synthesis (such as essential amino acids) and possible absorption. A thorough understanding of these segment-specific functions and their underlying unique molecular functions is essential for comprehensively grasping the overall digestive physiology and environmental adaptability of *Nezara viridula* [8,9,10,11].

Despite existing studies that have explored molecular-level differences between segments and their roles in digestive physiology [10,11], traditional research has primarily concentrated on individual genes, proteins, or specific regulatory pathways, lacking systematic analyses of comprehensive functional networks [12]. Furthermore, such studies often focus on particular physiological processes, consequently failing to fully elucidate the multi-layered functional distinctions and complex regulatory networks among midgut segments. Therefore, several core scientific questions remain unresolved, such as how each midgut segment coordinately responds to plant defense compounds, precisely regulates digestive and metabolic processes, and the molecular regulatory networks underpinning these biological phenomena [13]. Transcriptomics has emerged as a powerful tool for studying gene expression patterns, offering novel perspectives for elucidating segment-specific functional differentiation [14]. However, conventional transcriptomic analyses are typically limited to identifying differentially expressed genes, without delving into their functional networks and regulatory relationships [15]. Recently developed methods, such as pathway activity scoring systems and multi-layered functional enrichment analyses, offer promising new solutions for addressing these issues [16]; however, their application in the study of insect digestive physiology remains relatively limited.

To overcome the limitations of conventional analytical methods and fully leverage the power of integrated bioinformatics, this study employed a multi-level analytical strategy. These strategies offer distinct and unique advantages: they enable the efficient integration and profound interpretation of multidimensional data, surpassing the analytical scope of single tools and substantially reducing the time investment required for exploring and mastering various independent software packages prior to functional experimental validation. More importantly, this integrated approach provides nuanced, multi-layered biological insights, identifying not only characteristic genes but also their associated functional pathways, complex “multi-gene–multi-pathway” regulatory modules, and offering specific molecular interaction references through pathway maps and other visualizations. This methodology facilitates the construction of a more comprehensive and systemic understanding, and critically, allows subsequent functional experimental validation efforts to be precisely focused on the most promising targets and mechanisms. This research aims not only to deepen the fundamental understanding of insect digestive physiology but also to provide a robust scientific basis for the development of novel control strategies targeting the insect digestive system.

## 2. Materials and Methods

### 2.1. Transcriptome Data Preprocessing and Functional Annotation Pipeline

Leveraging the publicly available dataset GSE130097 from the NCBI GEO database [11], we systematically analyzed transcriptomic profiles from distinct midgut regions (M1–M3) of *Nezara viridula* under a maize-based diet. To ensure reliability, low-abundance transcripts were removed using an expression threshold (>10), and transcript identifiers were standardized to maintain consistency and integrity across samples.

Open reading frames (ORFs) were predicted using the R package ORFik [17], employing canonical start codons (ATG, TTG, CTG) and stop codons (TAA, TAG, TGA), with a minimum ORF length of 200 nucleotides. The longestORF option was used to prioritize the most biologically relevant ORF for each transcript [17,18]. Functional annotation was subsequently performed through orthology-based predictions [19]. Based on the annotated ORFs, mapping matrices were constructed, including gene-to-KEGG pathway relationships and pathway-to-functional-description references [20,21,22].

### 2.2. Pathway Activity and Segmental Transcriptomic Analysis

Principal component analysis (PCA) was performed to evaluate the clustering of different tissue samples and to assess the degree of heterogeneity among them. The resulting expression pattern differences across tissues were visualized using a PCA plot, which enhanced the clarity of inter-tissue variation. Subsequently, segment-specific gene modules were systematically identified by calculating the mean expression levels of each KO identifier in individual tissues (M1_mean, M2_mean, M3_mean), followed by filtering based on the coefficient of variation. This enabled the extraction of highly expressed, segment-enriched gene modules. Standardized heatmaps and trend plots were employed to visualize the expression patterns of these segment-specific genes in a comprehensive manner. Finally, KEGG enrichment analysis was conducted on the identified segment-enriched gene modules, offering preliminary insights into the link between gene expression and pathway-level functional specialization.

### 2.3. Advanced Visualization of Gene Set Enrichment Analysis

Based on the differential expression analysis, a ranked gene list was generated using log_2_ fold change values. The KEGG pathway database was refined and filtered for downstream gene set enrichment analysis (GSEA) on the ordered gene list. A minimum gene set size (minGSSize = 5) was applied, and the results were ranked according to the normalized enrichment score (NES) [23,24,25]. Visualization of both pathway collections and individual enriched pathways was carried out using the R packages Metatrx and GseaVis [26], enabling a more informative and interpretable representation of the enrichment patterns.

### 2.4. Topological Mapping of Gene Interaction Networks

A multi-layered pathway enrichment network visualization system was established using the aPEAR tool [27]. Node colors and legend scales were assigned according to *p*-values and gene set sizes, enabling intuitive visualization of functional associations among pathways and facilitating the identification of key functional modules and regulatory hubs. Finally, the significantly enriched pathways identified in GSEA [28] were intersected with the regulatory core pathways filtered in this step, and core gene names were annotated for the intersected pathways.

### 2.5. Pathway Activity Differentiation Analysis Across Tissues Using ReporterScore

A data preprocessing pipeline was developed to compare three sample groups (M1 vs. M2, M1 vs. M3, and M2 vs. M3). Sample grouping matrices were constructed based on critical variables such as tissue type, diet, and developmental stage. A pathway activity scoring system was then established using the ReporterScore package, which was applied to differential expression analysis across different tissue regions (M1, M2, M3). To achieve a more holistic understanding of pathway dynamics beyond individual gene changes, this study employed the ReporterScore method. This approach offers a pathway-centric perspective by scoring pathway activity based on feature *p*-values, thereby sensitively capturing subtle yet coordinated shifts in gene expression within pathways, without reliance on pre-set differential expression (DE) cut-off thresholds. Pathway significance was assessed via the ReporterScore algorithm [29]. For each pathway, Z-scores and *p*-values were calculated, and multiple testing corrections were performed to ensure result reliability. This approach enabled a more systematic functional analysis, transitioning from gene-level to pathway-level insights.

## 3. Results

### 3.1. PCA of Different Midgut Regions in Nezara viridula

Principal component analysis (PCA) revealed significant differences in transcriptome levels among different midgut regions of *Nezara viridula*. The first principal component (PC1) accounted for 54.7% of the variance, while the second principal component (PC2) accounted for 20.7%, with a cumulative contribution rate of 75.4%. Along the PC1 axis, the digestive tract tissues showed a clear distribution pattern, arranged from left to right as M3, M2 and M1, indicating segment-specific gene expression patterns. Meanwhile, biological replicate samples (rep1, rep2 and rep3) of each tissue exhibited good clustering, confirming the reliable reproducibility of the data. These results demonstrate significant differences in transcriptome levels among midgut regions (Figure S1).

### 3.2. Enrichment Analysis of Highly Expressed Genes Specific to Different Midgut Regions

**Figure 1:**
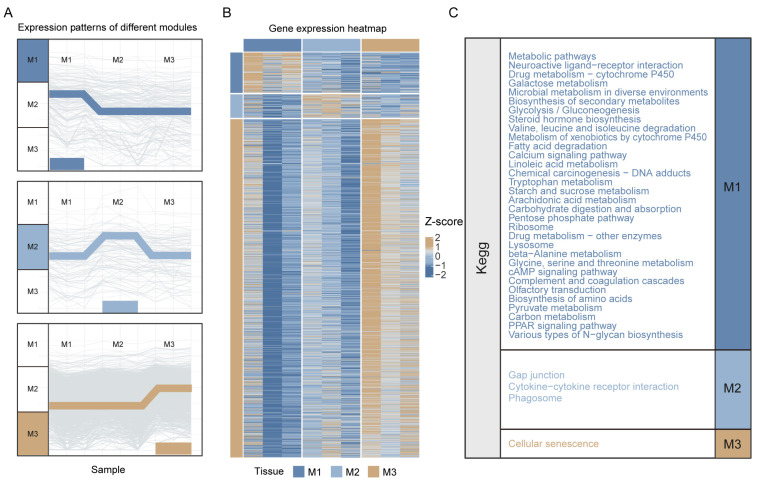
Heatmap of gene expression and pathway enrichment results in different midgut regions (M1, M2, M3). (**A**) Genes with the highest expression in each midgut segment (M1, M2, M3) were grouped accordingly. Light background lines represent the expression trends of all genes within each group across the segments, while the bold line highlights the overall expression trend of the group, intuitively illustrating the expression changes of these genes and the segment in which their expression reaches its maximum. (**B**) Heatmap of differentially expressed genes across M1, M2, and M3, where each row is a gene and each column is a sample. Colors represent Z-scores of log2-transformed expression values (brown: high, blue: low). (**C**) KEGG pathways significantly enriched in each segment of the midgut, grouped and color-coded by segment.

To systematically analyze the functional differentiation characteristics of different midgut regions (M1, M2, M3) in *Nezara viridula* fed on maize, we performed comprehensive transcriptome analysis on these regions. Gene expression profiling revealed three significant segment-specific high-expression patterns, which were visualized through gene expression trend graphs (Figure 1A) and heatmaps (Figure 1B). The heatmap used a color gradient ranging from low expression (blue, −2) to high expression (brown, 2), clearly showing the differences in expression intensity of various genes across different tissues.

KEGG pathway enrichment analysis further elucidated the functional differentiation characteristics of these midgut segments. The M1 segment was found to be significantly enriched in a variety of core metabolic pathways, broadly encompassing major carbohydrate metabolism, lipid metabolism, and amino acid metabolism processes. Furthermore, the M1 region also exhibited enrichment in the biosynthesis of secondary metabolites, drug metabolism, and key signal transduction pathways (e.g., calcium signaling, cAMP signaling, and PPAR signaling pathways). In comparison, the M2 segment showed enrichment in a smaller number of pathways, primarily concentrated in functional categories such as cell junction and communication, immune-related functions, and phagocytosis. Enrichment analysis of the M3 segment indicated its close association with the cellular senescence pathway. Although the number of enriched pathways in this region was relatively limited, this finding is of clear biological significance, suggesting that the M3 segment may be specialized for cell renewal and the final processing of digested substances (Figure 1C).

### 3.3. Construction of KEGG Pathway Network and Systematic Identification of Functional Modules in Nezara viridula

To systematically characterize the functional specialization among midgut regions (M1, M2, M3), we conducted an integrative multi-omics analysis combining (1) differential gene expression profiling; (2) Gene Set Enrichment Analysis (GSEA); (3) KEGG pathway network reconstruction; and (4) functional module identification. This multi-dimensional analytical strategy not only identified characteristic pathways for each region but also revealed functional interconnections among these pathways and their synergistic mechanisms in insect digestive physiology. Notably, through the intersection validation of GSEA and aPEAR analysis results, we were able to reliably identify key functional pathways and their core regulatory genes with high confidence. These findings provide molecular-level evidence for understanding the functional compartmentalization of the *Nezara viridula* midgut.

**Figure 2:**
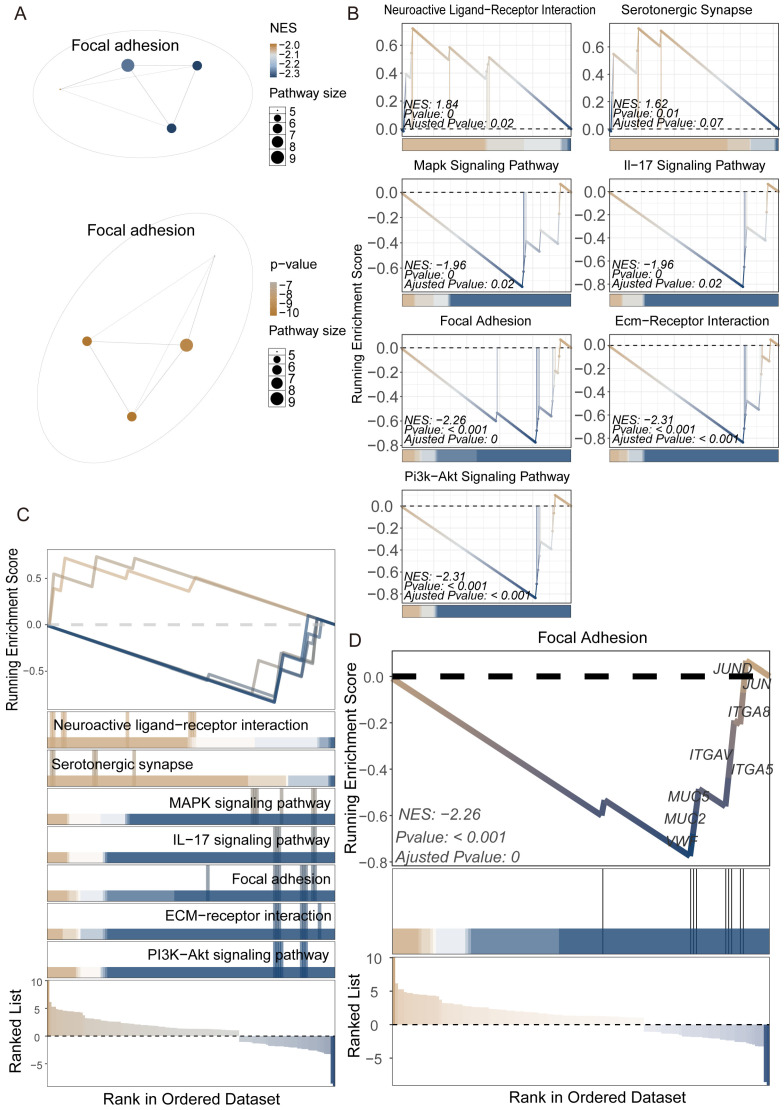
Differential metabolic and signaling pathway analysis between M1 and M2 tissues using GSEA and aPEAR approaches. (**A**) Pathway network visualization of significantly enriched pathways between M1 and M2. Node color represents normalized enrichment score (NES, top) or *p*-value (bottom), and node size indicates pathway size. (**B**) GSEA enrichment plots for representative pathways. (**C**) Summary of GSEA running enrichment scores for the top significant pathways. The x-axis shows the rank in the ordered gene set, and the y-axis shows the running enrichment score. (**D**) Detailed GSEA plot for the focal adhesion pathway, highlighting the leading-edge genes contributing to the enrichment signal. The NES, *p*-value, and adjusted *p*-value are indicated.

The functional differences between M1 and M2 tissues are shown in Figure 2. This figure integrates multiple visualization methods to demonstrate the pathway enrichment comparison between M1 and M2. The aPEAR network diagram constructs a functional association network of the focal adhesion pathway through topological algorithms, with blue nodes representing pathways upregulated in M2. The lower network diagram shows the significance of the focal adhesion pathway from a *p*-value perspective, where the progression from orange to grey indicates the degree of significance (Figure 2A). Figure 2C displays the ranking of pathways in the gene ranking list, where neuroactive ligand–receptor interaction and serotonergic synapse are upregulated in M1, while the MAPK signaling pathway, focal adhesion, ECM–receptor interaction, and PI3K-Akt signaling pathway are upregulated in M2. Figure 2B details the enrichment of seven key pathways: neuroactive ligand–receptor interaction (NES = 1.84), serotonergic synapse (NES = 1.62), MAPK signaling pathway (NES = −1.96), IL-17 signaling pathway (NES = −1.96), focal adhesion (NES = −2.26), ECM–receptor interaction (NES = −2.31), and PI3K-Akt signaling pathway (NES = −2.31). Figure 2D specifically shows the focal adhesion pathway (NES = −2.26, *p* < 0.001), with related genes such as *ITGA8*, *ITGAV*, *ITGA5*, *MUC5*, and *MUC2* mainly distributed in the M2 upregulated region. These results indicate that M1 tissue shows significant activity in neural signal transduction and inflammatory response-related pathways, while M2 tissue is enriched in cell adhesion, extracellular matrix interaction, and growth signaling pathways. Consequently, the M1 segment likely undertakes key sensory, regulatory, and initial response functions at the anterior of the midgut, whereas the M2 segment appears specialized for structural maintenance and intercellular communication, establishing a complex and ordered physiological foundation for the anterior midgut.

**Figure 3:**
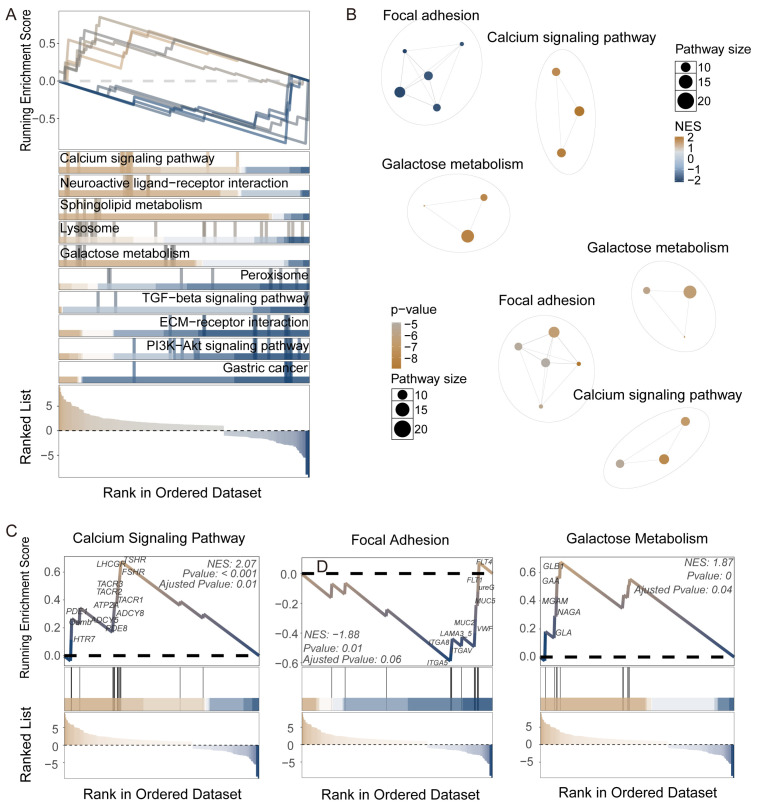
Differential analysis of metabolism and signaling pathways between M1 and M3 tissues based on GSEA and aPEAR. (**A**) Summary of GSEA running enrichment scores for the most significant pathways distinguishing M1 and M3 segments. (**B**) Network visualization of representative enriched pathways. Node color represents normalized enrichment score (NES, top) or *p*-value (bottom), and node size indicates pathway size. (**C**) GSEA enrichment plots for three key pathways. The NES, *p*-value, and adjusted *p*-value are indicated in each panel, and leading-edge genes are labeled.

Figure 3 and Figure S2 present the results of metabolic and signaling pathway differential analysis between M1 and M3 tissues based on combined GSEA and aPEAR analysis. Figure 3A is a GSEA ranking plot showing the distribution of various pathways in the gene ranking list, with a calcium signaling pathway, neuroactive ligand–receptor interaction, sphingolipid metabolism, lysosome, and galactose metabolism significantly upregulated in M1, while peroxisome, TGF-beta signaling pathway, ECM–receptor interaction, and PI3K-Akt signaling pathway were significantly upregulated in M3. In Figure 3B, orange nodes represent pathways upregulated in M1, such as the calcium signaling pathway and galactose metabolism. Figure 3C details the enrichment of three key pathways after intersection: the calcium signaling pathway (NES = 2.07, *p* < 0.001) was significantly upregulated in M1, with related genes including *HTR7*, *Oamb*, *PDE1*, *PDE8*, *TACR2*, *TACR3*, *LHCGR*, *TSHR*, etc.; focal adhesion (NES = −1.88, *p* = 0.01) was upregulated in M3, with related genes including *FLT4*, *FLT1*, *ureG*, *MUC5*, *MUC2*, *LAMA3_5*, *ITGA8*, *ITGA5*, and *ITGAV*; galactose metabolism (NES = 1.87, *p* = 0) was upregulated in M1, with related genes including *GLA*, *NAGA*, *MGAM*, *GAA*, and *GLB1*. These results indicate that M1 tissues show significant activity in calcium signal transduction, neural signaling, and specific metabolic pathways, while M3 tissues are enriched in cell adhesion, extracellular matrix interaction, and growth signaling pathways. This array of molecular characteristics clearly delineates M1 as a hub for early digestive processes and sensory–regulatory functions, contrasting with M3’s specialization in posterior midgut tasks such as cellular renewal and final nutrient processing, thereby establishing a distinct functional division of labor.

Figure S3 shows the results of differential analysis of metabolism and signaling pathways between M2 and M3 tissues based on GSEA and aPEAR (the two methods did not identify any intersection pathways). The enrichment score curve in the upper left of Figure S3A shows the distribution trend of pathways in the gene ranking list, with brown curves representing pathways upregulated in M2 and blue curves representing pathways upregulated in M3. Figure S3B details the enrichment of these three key pathways: the lysosome pathway is significantly upregulated in M2 (NES = 1.82, *p* = 0.01), with the enrichment score curve showing a distinct upward trend; the TGF-beta signaling pathway is upregulated in M3 (NES = −1.73, *p* = 0.01), with the enrichment score curve showing a downward trend; similarly, the arginine and proline metabolism pathway is also upregulated in M3 (NES = −1.78, *p* = 0.01), with the enrichment score curve also showing a downward trend. Due to the limited number of enriched pathways in this module, aPEAR analysis did not generate an associated pathway network diagram. These results suggest that M2 tissues may be more active in lysosomal function, while M3 tissues show significant activity in TGF-beta signal transduction and specific amino acid metabolism, revealing the differential molecular functional characteristics between M2 and M3 tissues.

### 3.4. Gene Regulatory System Analysis and GRSA Visualization

Previous analyses have revealed functional differences between different segments from multiple perspectives. To further quantify these differences and ensure the reliability of the results, we adopted a system analysis strategy based on ReporterScore. This method not only rigorously evaluates pathway significance through Z-scores and corrected *p*-values, but also performs hierarchical clustering of functions across multiple levels (level 1–3) of the KEGG database, and combines KEGG pathway maps to intuitively present the precise locations of differentially expressed genes, thus providing more rigorous and systematic evidence for functional differences.

**Figure 4:**
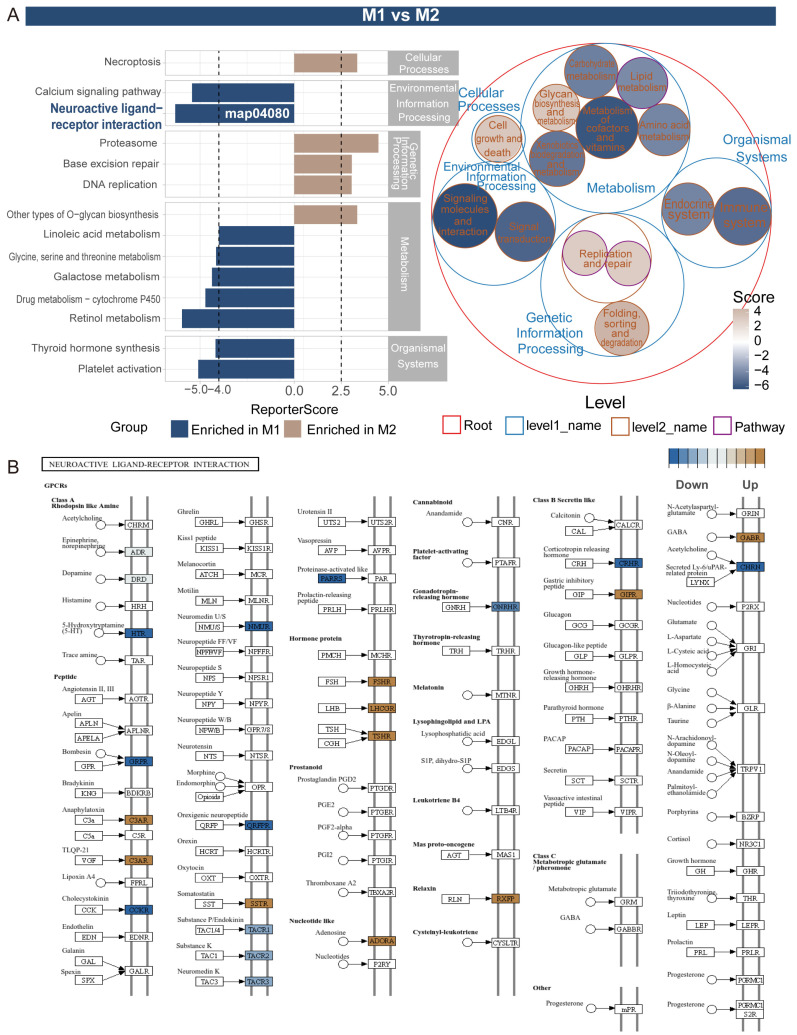
GRSA analysis of M1 and M2 midgut regions. (**A**) ReporterScore bar plot of significantly enriched KEGG pathways (brown: enriched in M2, blue: enriched in M1). The bubble plot on the right summarizes higher-level functional categories above the pathway level, with bubble size representing the ReporterScore for each category and color indicating enrichment in M1 or M2. (**B**) Detailed map of the neuroactive ligand–receptor interaction pathway, highlighting differentially expressed genes. The comparison of M1 vs. M2 reveals significant differences in pathway enrichment. In the results, neuroactive ligand–receptor interaction is highly enriched in M1, along with pathways such as drug metabolism; whereas M2 is enriched in pathways related to cell death and repair, such asNecroptosis, proteasome, and DNA replication. The bubble chart further illustrates the hierarchical structure and enrichment levels of these pathways (**A**).

Through visualization analysis of the KEGG neuroactive ligand–receptor interaction pathway map, we observed significant differential expression of multiple receptors related to insect neuroendocrine regulation. Among them, the tachykinin receptor family (tachykinin receptor, *TACR1-3*) shows significant downregulation (blue), which may represent key molecules regulating insect midgut peristalsis and digestive enzyme secretion; in terms of digestive activity regulation, the significant downregulation (blue) of 5-hydroxytryptamine receptor (*HTR*) reflects changes in neural regulation of midgut digestive activity, while the upregulation (brown) of adenosine receptor (*ADORA*) may be related to energy metabolism regulation during digestion. These changes in the expression patterns of key receptors reveal the differences in digestive function regulation between the M1 and M2 segments of the insect midgut, reflecting the functional differentiation characteristics of different midgut segments (Figure 4B and Figure S4).

**Figure 5:**
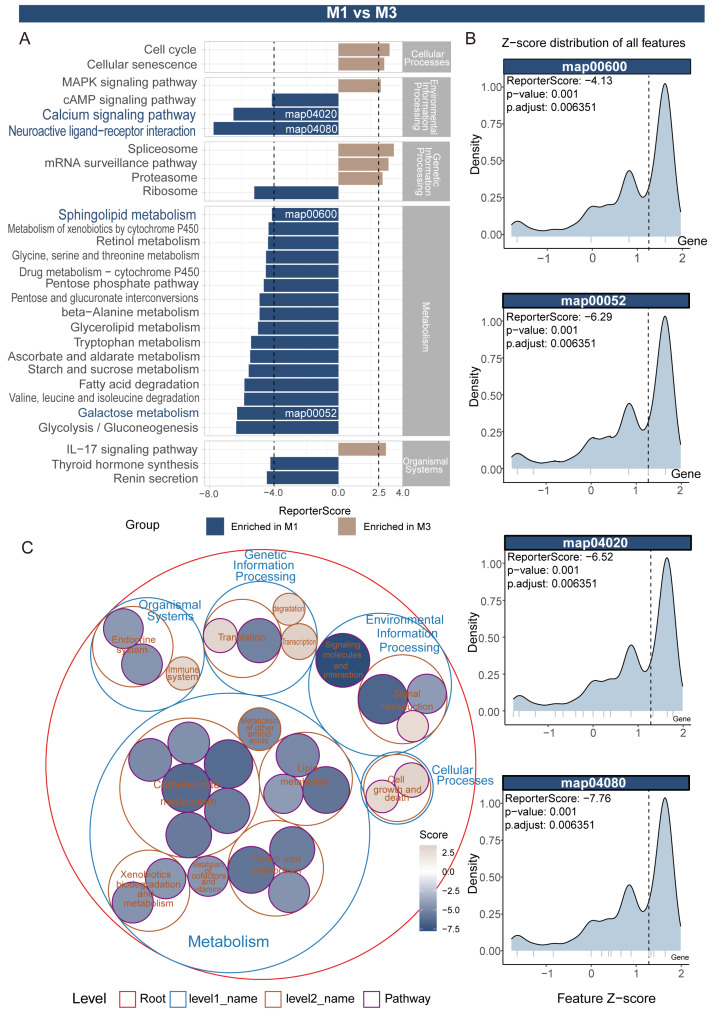
GRSA analysis of M1 and M3 midgut regions. (**A**) ReporterScore bar plot showing significantly enriched KEGG pathways between M1 and M3 (blue: enriched in M1, brown: enriched in M3), with pathway categories indicated on the right. (**B**) Density plots of gene Z-score distributions for representative pathways. (**C**) Bubble plot summarizing higher-level functional categories above the pathway level; bubble size represents the ReporterScore for each category, and color indicates enrichment direction (blue: M1, brown: M3).

The M1 vs. M3 comparison shows four key pathways significantly enriched in M1: neuroactive ligand–receptor interaction (map04080, ReporterScore −7.76), calcium signaling pathway (map04020, ReporterScore −6.52), sphingolipid metabolism (map00600, ReporterScore −4.13), and galactose metabolism (map00052, ReporterScore −6.29), all with significant *p*-adjusted values (0.006351). In contrast, M3 is enriched in pathways related to cell cycle, cellular senescence, and mRNA surveillance pathway (Figure 5).

**Figure 6:**
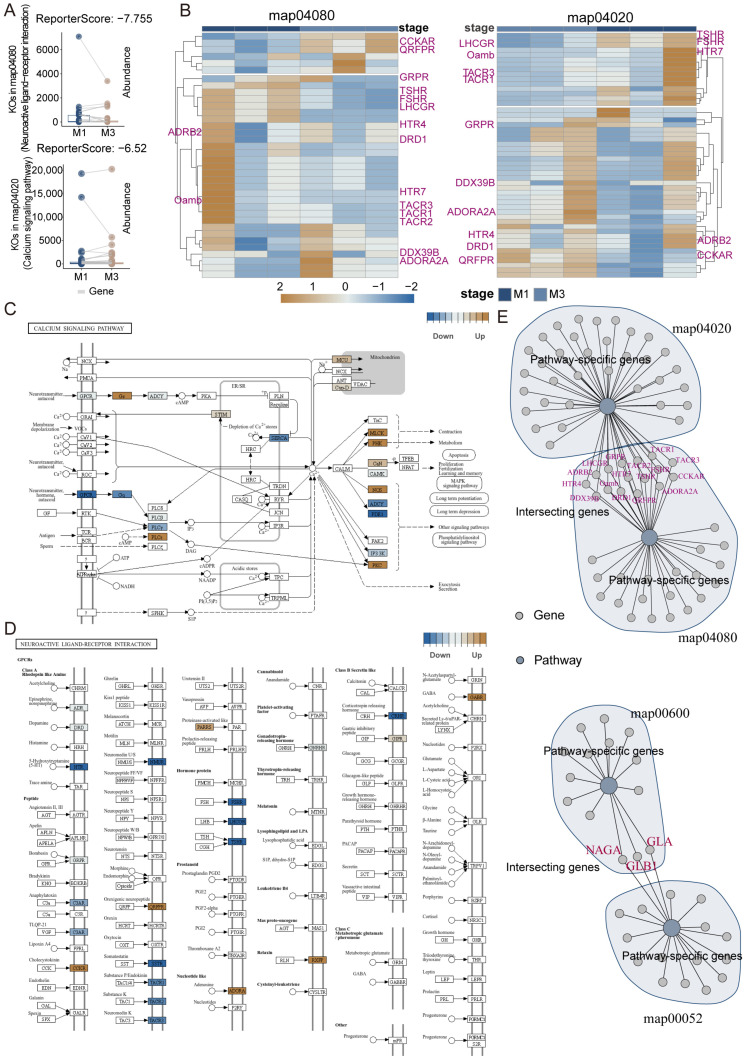
GRSA analysis of M1 and M3 (neuroactive ligand–receptor interaction, calcium signaling pathway): (**A**) abundance of pathway-specific genes in representative KEGG pathways. (**B**) Heatmaps showing expression patterns (Z-scores) of genes in the neuroactive ligand–receptor interaction (map04080) and calcium signaling (map04020) pathways across M1 and M3. (**C**,**D**) KEGG pathway diagrams highlighting differentially expressed genes (blue: downregulated in M3, brown: upregulated in M3). Arrows indicate the direction of signal transduction or molecular interaction between ligands and receptors, following the conventions of the KEGG pathway map. (**E**) Network visualization of pathway-specific and intersecting genes among the four representative pathways; pathway-specific genes are shown in gray, and intersecting genes are labeled in magenta.

In environmental information processing pathway analysis, the integrated analysis of the calcium signaling pathway (map04020) and neuroactive ligand–receptor interaction pathway (map04080) reveals a detailed molecular network regulating digestive functions. In the calcium signaling pathway, G protein-coupled receptors (GPCRs) are significantly downregulated (blue), indicating potentially weakened reception of neurotransmitter and hormone signals; the phospholipase C family (PLC) shows differential expression, with *PLCγ* downregulated and *PLCε* upregulated (brown), suggesting selective activation of different signal transduction pathways. In calcium regulation, sarco/endoplasmic reticulum Ca^2+^-ATPase (*SERCA*) is significantly downregulated, potentially affecting intracellular calcium store filling, while calmodulin (*CALM*) serves as a central regulatory molecule connecting multiple downstream effector pathways. Analysis of the neuroactive ligand–receptor interaction pathway (map04080) reveals differential expression patterns of key receptors. Among monoamine receptors, 5-hydroxytryptamine receptor (*HTR*) is significantly downregulated (blue), while GABA receptor (*GABR*) is upregulated (brown). In the neuropeptide receptor system, multiple receptors including tachykinin receptor (*TACR1/2/3*) and gonadotropin-releasing hormone receptor (*GNRHR*) show downregulation trends, whereas cholecystokinin receptor (*CCKR*), relaxin receptor (*RXFP*), and pyroglutamylated RFamide peptide receptor (*QRFPR*) show upregulation trends, which are closely related to feeding behavior and digestive function regulation. The upregulation of adenosine receptor (*ADORA*) may be related to energy metabolism (Figure 6).

In the metabolism-related pathway analysis, the galactose metabolism pathway shown in Figure S5 reveals the upregulation of key enzymes EC 5.1.3.3 (aldose 1-epimerase) and EC 2.7.1.1/2 (hexokinase/glucokinase), promoting the conversion of galactose to UDP-galactose and the phosphorylation of glucose, while the downregulation of hydrolase EC 3.2.1.22 (α-galactosidase) may slow down polysaccharide degradation. This pattern indicates that the insect gut is prioritizing the channeling of monosaccharides into the UDP-sugar synthesis pathway rather than energy production pathways, possibly for glycoprotein and glycolipid modification and maintenance of gut barrier function, while also potentially providing precursors for the biosynthesis of other structural components. Meanwhile, in the sphingolipid metabolism pathway, the upregulation of EC 2.3.1.50 (serine C-palmitoyltransferase), CERS (ceramide synthase), and EC 3.1.3.4 (phosphatidate phosphatase) enhances ceramide synthesis or dephosphorylation, while the downregulation of EC 2.4.1.47 (N-acylsphingosine galactosyltransferase) reduces galactosylceramide synthesis (Figure S6).

In the M2 vs. M3 comparison, M2 is enriched in various metabolism-related pathways, including ribosome, amino acid metabolism (phenylalanine metabolism, tryptophan metabolism), sphingolipid metabolism, xenobiotic metabolism (metabolism of xenobiotics by cytochrome P450), beta-alanine metabolism, fatty acid degradation, and carbohydrate digestion and absorption. In contrast, M3 is enriched in the mRNA surveillance pathway and cytosolic DNA-sensing pathway. The bubble chart clearly illustrates the hierarchical relationships of these metabolic pathways, with “Metabolism” as the main centre, radiating into multiple specific metabolic processes, revealing the differences in metabolic functions between M2 and M3 (Figure S7).

## 4. Discussion

The KEGG pathway enrichment analysis of different segments of *Nezara viridula* midgut reveals distinct functional compartmentalization. This partitioning likely reflects the evolutionary adaptations and functional specificity of the insect digestive tract. The enrichment of numerous metabolic pathways in the M1 segment suggests that it serves as the primary metabolic hub of the insect midgut. This segment’s enrichment in carbohydrate, lipid, and amino acid metabolic pathways indicates a central role in initial food digestion and the preliminary processing of nutrients. Additionally, the enrichment of drug metabolism pathways (cytochrome P450-related) in the M1 segment highlights its potential role in detoxification, which is critical for handling plant-derived secondary metabolites and defensive compounds. The presence of calcium and cAMP signaling pathways points to fine-tuned regulation of digestive enzyme secretion, ensuring the digestion process can dynamically adjust to the composition of food.

The M2 segment’s pathway enrichment profile suggests a role in intercellular communication and immune defense. The enrichment of the gap junction pathway indicates the presence of a tight communication network among M2 cells, which is important for coordinating digestive activities and maintaining epithelial barrier integrity. Meanwhile, the enrichment of cytokine–cytokine receptor interactions and phagosome pathways suggests that M2 plays a key role in the insect gut’s immune defense, serving as an important barrier against foodborne pathogens.

The association between the M3 segment and the cellular senescence pathway carries profound biological significance. This connection may indicate a high turnover rate of epithelial cells in the M3 region, with cells undergoing programmed senescence and renewal to maintain the integrity and functionality of the intestinal epithelium. As the posterior section of the midgut, M3 likely focuses on final nutrient absorption and waste processing in preparation for excretion.

To ensure the scientific rigor and applicability of our pathway enrichment analysis, we excluded disease-related pathways irrelevant to insects prior to analysis, and employed gene set enrichment methods such as GSEA and GSRA, which focus on the overall expression trends of gene sets. This approach effectively reduces annotation errors and human bias. The presence of certain mammalian-related pathways in our results likely reflects the evolutionary conservation of fundamental biological processes, and their enrichment in insects suggests that these mechanisms may also play important roles in insect physiology.

Building on these findings, a multilayer analysis using ReporterScore was employed to further explore functional differences across segments. ReporterScore supports functional clustering at multiple KEGG pathway levels (level 1, level 2, and level 3), enabling differential gene annotation from basic functions to higher-level functional categories. This approach constructed a comprehensive functional hierarchy network, while the combination of pathway topology and KEGG pathway map visualization allowed for precise localization of key differentially expressed genes within metabolic pathways, offering a clear representation of their potential regulatory roles.

Notably, comparisons between M1 and M2 segments revealed a high enrichment of the neuroactive ligand–receptor interaction pathway in M1, suggesting that the anterior midgut is a major regulatory center for digestive activities. Pathway visualization showed significantly higher expression of *TACR1-3* and *HTR* in M1 (marked in blue), while *ADORA* was more highly expressed in M2 (marked in brown). This region-specific expression pattern correlates with the region-specific distribution of neuropeptide systems observed in previous studies, such as those by Veenstra et al. (2008) on the *Drosophila* midgut, suggesting a functional relationship between ligand distribution and receptor expression patterns [30]. Additionally, Audsley and Weaver (2009) discussed neuropeptide regulation of insect feeding behavior, including the distribution and functions of various neuroactive substances in the insect gut, which aligns with the discovery of *TACR* receptors in this study [31]. Furthermore, neuropeptides and their receptors (such as GPCRs) in the insect gut play crucial roles in regulating gut motility, digestion, and energy metabolism [32], while adenosine receptors (*ADORA*) can activate cAMP and calcium signaling pathways, contributing to the maintenance of gut homeostasis [33]. These mechanisms provide a molecular basis for the functional differentiation and adaptability of different gut regions, and the region-specific distribution of neuroendocrine regulatory networks may be a key mechanism by which *Nezara viridula* adapts to different food components and dynamically adjusts its digestive strategies.

Comparisons between M1 and M3 segments further revealed a functional gradient along the midgut. Enrichment of calcium signaling and neuroactive ligand–receptor interaction pathways in M1 suggested an integrated signaling network, where GPCRs and *PLCγ* showed higher expression in M1 (marked in blue), while *PLCε* had higher expression in M3 (marked in brown). This differential distribution of signaling molecules forms a complex regulatory network that may support the specialized physiological functions of different midgut segments. For instance, in the galactose metabolism pathway, EC 5.1.3.3 (aldose 1-epimerase) and EC 2.7.1.1/2 (hexokinase/glucokinase) were more highly expressed in M3 (brown markers), while EC 3.2.1.22 (α-galactosidase) was more highly expressed in M1 (blue markers), suggesting that the posterior midgut may favor UDP-sugar biosynthesis pathways, whereas the anterior midgut may focus on polysaccharide breakdown. These findings are consistent with the region-specific metabolic specialization model proposed by Terra and Ferreira (2012), which demonstrated distinct metabolic roles for different midgut segments in insects [13]. Moreover, the enrichment of cell cycle and cellular senescence pathways in M3 indicates a focus on cell renewal and tissue maintenance in the posterior midgut, consistent with the model of periodic epithelial renewal in the insect midgut proposed by Hakim et al. (2010) [34]. This serves as the cellular foundation for maintaining gut health and defensive functions.

Consequently, the present investigation deepens our comprehension of intricate functional differentiation within the hemipteran insect digestive tract. Elucidated region-specific molecular signatures of M1–M3 midgut segments in *Nezara viridula*—a pivotal role in nutrient metabolism and neural signaling for the M1 region, specialization in cell communication and immune defense for the M2 region, and a predominant function in cell renewal and tissue maintenance for the M3 region —furnish a valuable molecular blueprint and theoretical underpinning for developing environmentally benign, highly targeted novel pest control strategies. Modulating key genes within identified segment-specific pathways (for example, TACR, HTR, GLA, and NAGA in the M1 region) offers promising new avenues for precise control of agricultural pests exhibiting similar physiological structures.

## 5. Conclusions

The multi-segment transcriptomic analytical pipeline and cross-validation methodology established in this study not only provide fundamental insights into the molecular mechanisms underlying digestive physiology in hemipteran insects, but also offer an adaptable strategy for the functional characterization of other biological systems. These findings outline clear directions for future experimental investigations: (1) validation of segment-specific molecular mechanisms; (2) exploration of the interaction between nutrient metabolism and neural regulation; and (3) identification of potential targets for pest control. The comprehensive analytical framework represents a critical resource for advancing research in insect physiology, evolutionary biology, and pest management.

## Data Availability

The transcriptomic data analyzed in this study were obtained from the publicly available GEO dataset GSE130097, which includes RNA-seq data from *Nezara viridula* midgut sections (M1–M3).

## Supplementary Materials

The following supporting information can be downloaded at: https://www.mdpi.com/article/10.3390/insects16060634/s1, Figure S1: PCA of Corn Samples; Figure S2: GSEA analysis of differentially expressed genes between M1 and M3 tissues; Figure S3: Differential analysis of metabolic and signaling pathways between M2 and M3 tissues using GSEA and aPEAR; Figure S4: Differential gene expression in neuroactive ligand-receptor interaction pathways; Figure S5: Pathway diagram of galactose metabolism; Figure S6: Pathway diagram of sphingolipid metabolism; Figure S7: Comparative analysis of M2 and M3 tissues by GRSA.
