# Supplementary material for: Functional Differentiation Reconfiguration in the Midgut of Nezara viridula (Hemiptera: Pentatomidae) Based on Transcriptomics: Multilayer Enrichment Analysis and Topological Network Interpretation
Source: Insects. 2025 Jun 16;16(6):634. doi: 10.3390/insects16060634 (PMC12193252; doi:10.3390/insects16060634)
Supplement: Supplementary file 1 [file insects-16-00634-s001.zip › insects-3635387-supplementary.pdf]

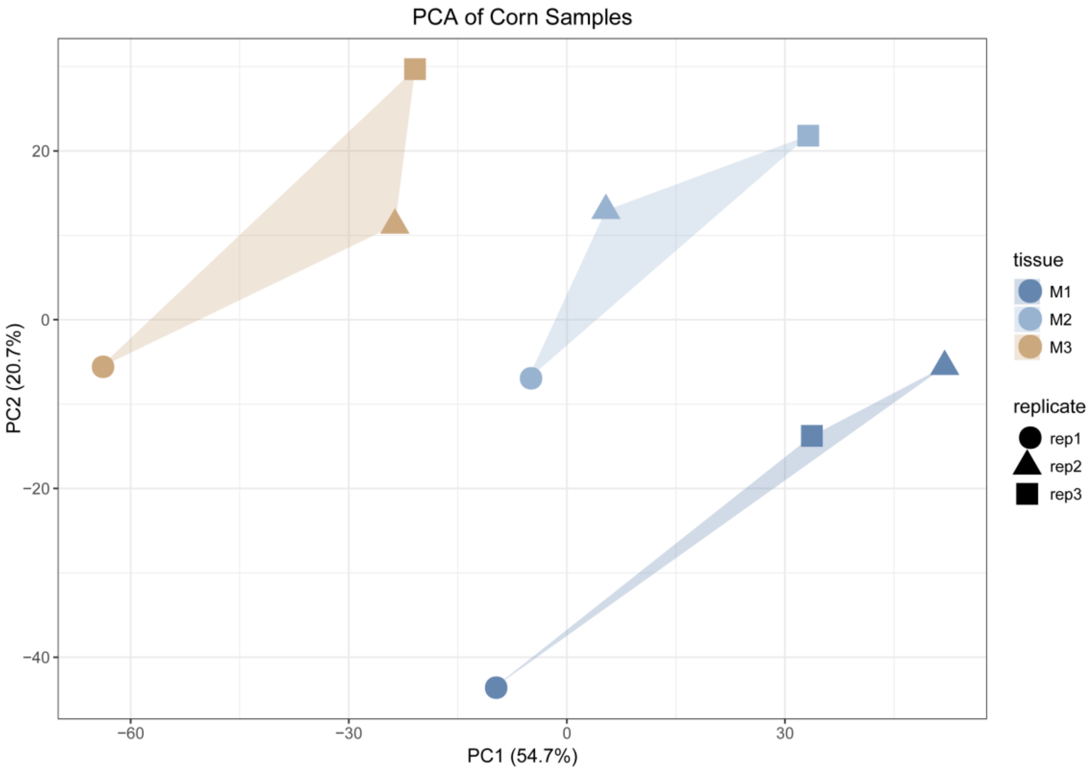

Figure S1. Principal component analysis of transcriptomes from distinct midgut regions (M1, M2, M3) in *Nezara viridula*

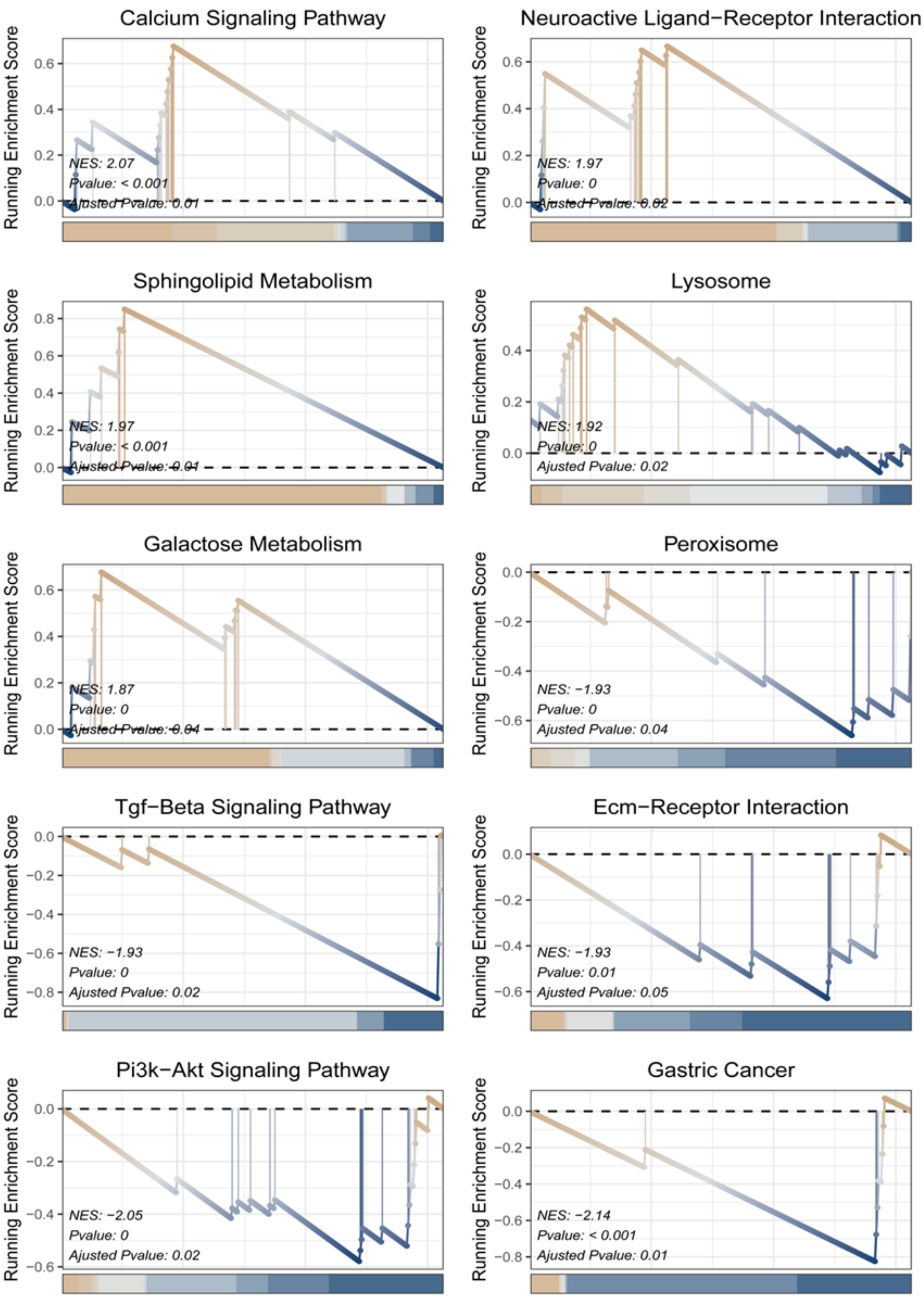

Figure S2. GSEA analysis of differentially expressed genes between M1 and M3 tissues.

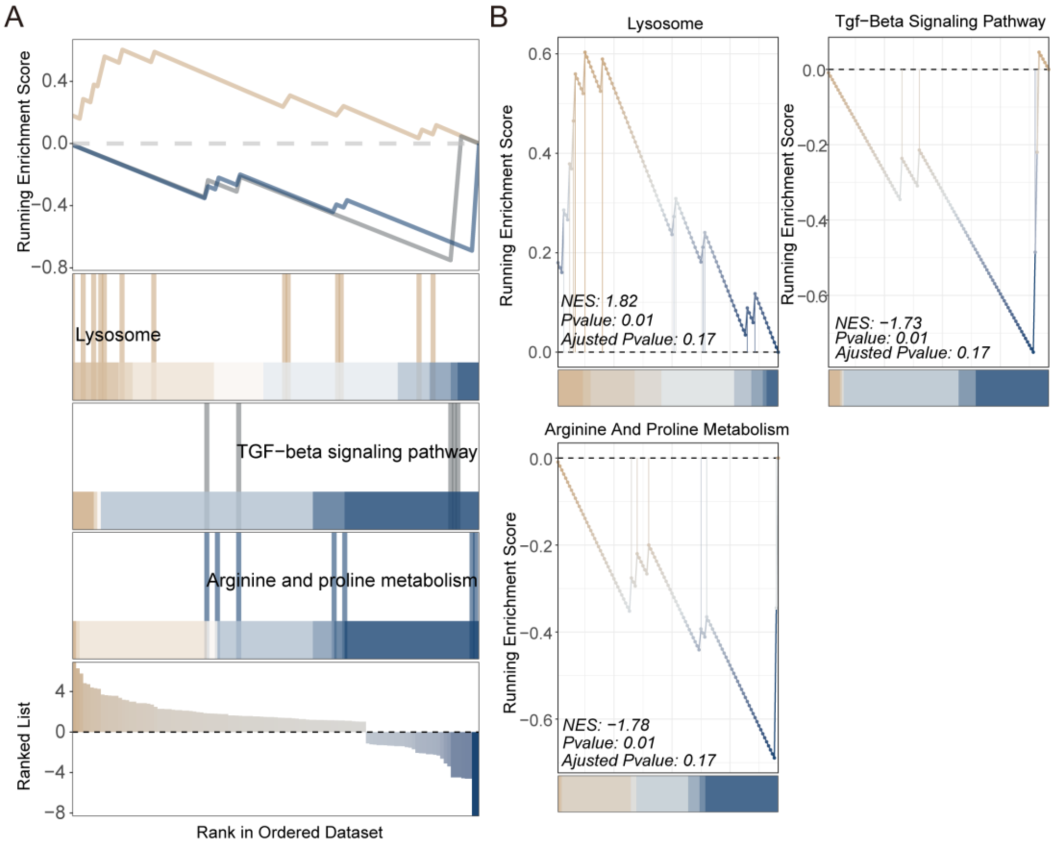

Figure S3. Differential analysis of metabolic and signaling pathways between M2 and M3 tissues using GSEA and aPEAR.

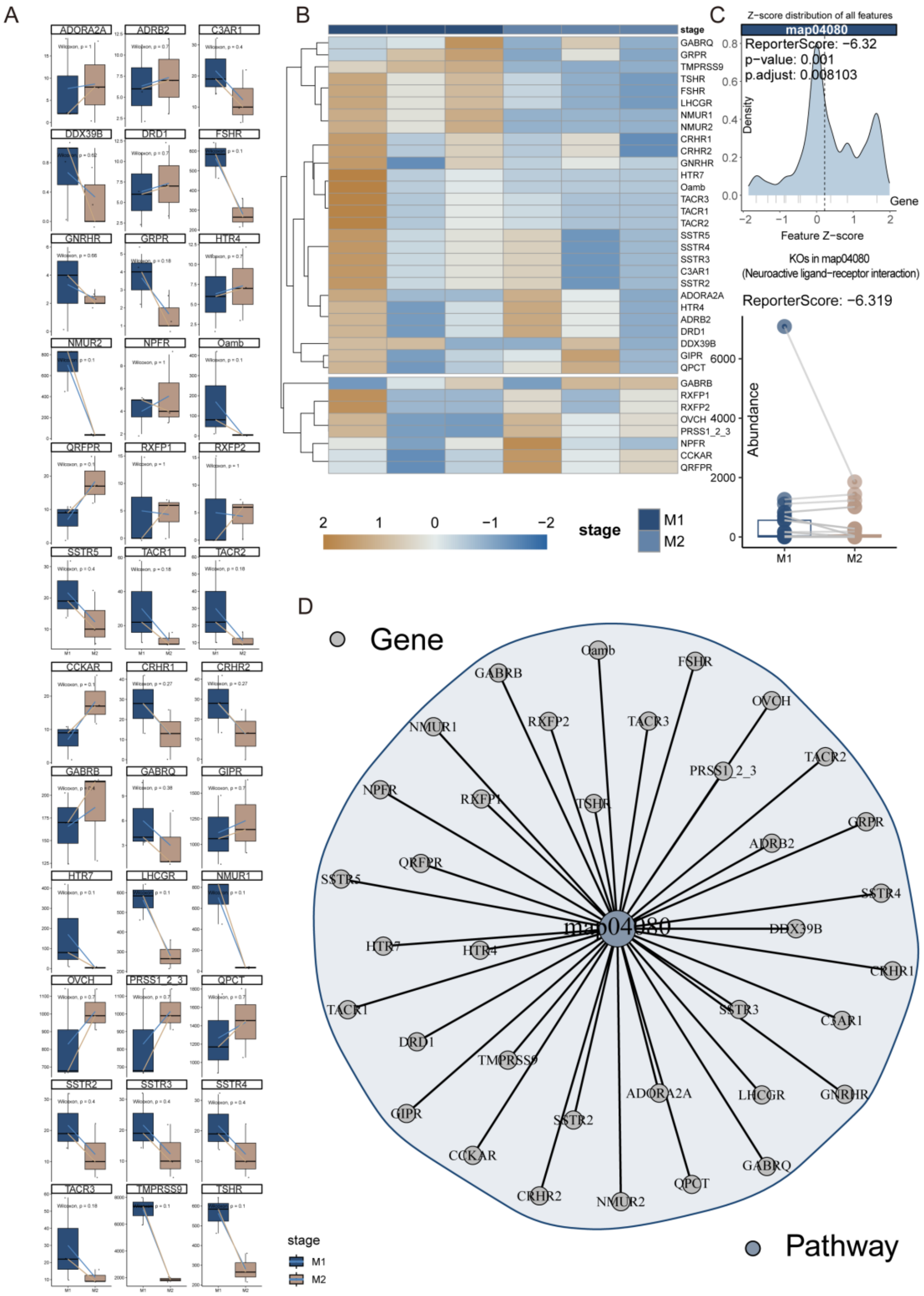

Figure S4. Differential gene expression in neuroactive ligand-receptor interaction pathways

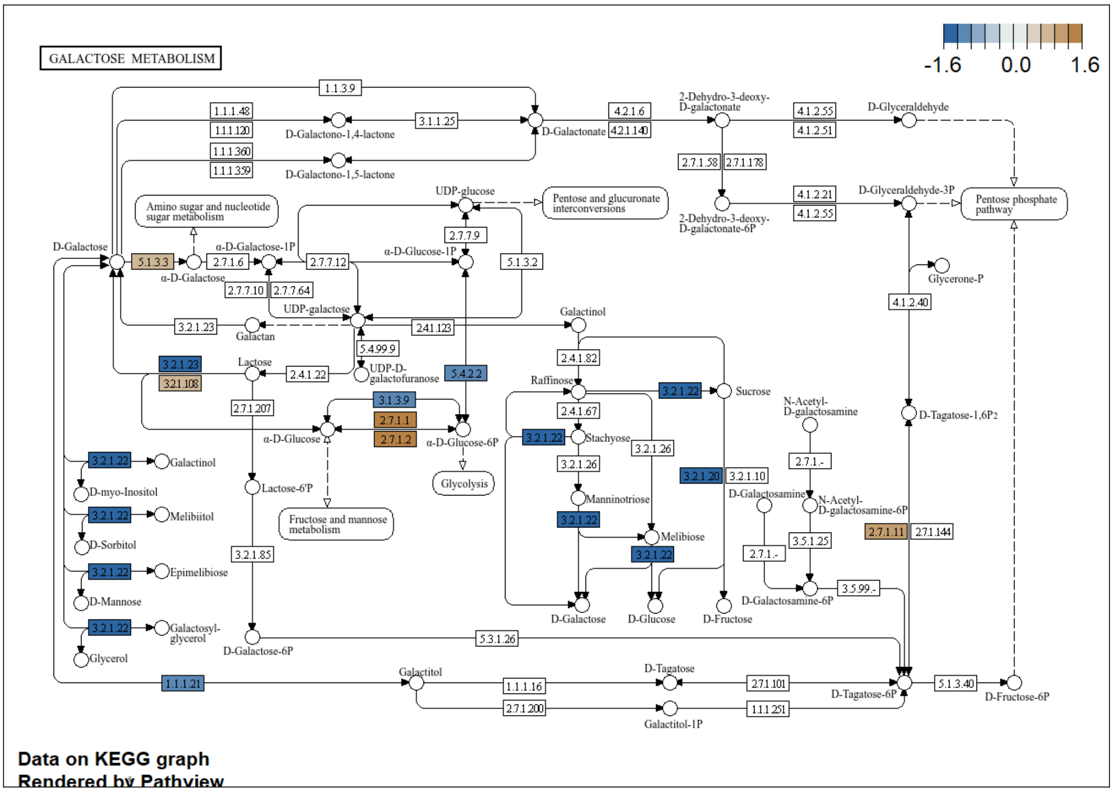

Figure S5. Pathway diagram of galactose metabolism

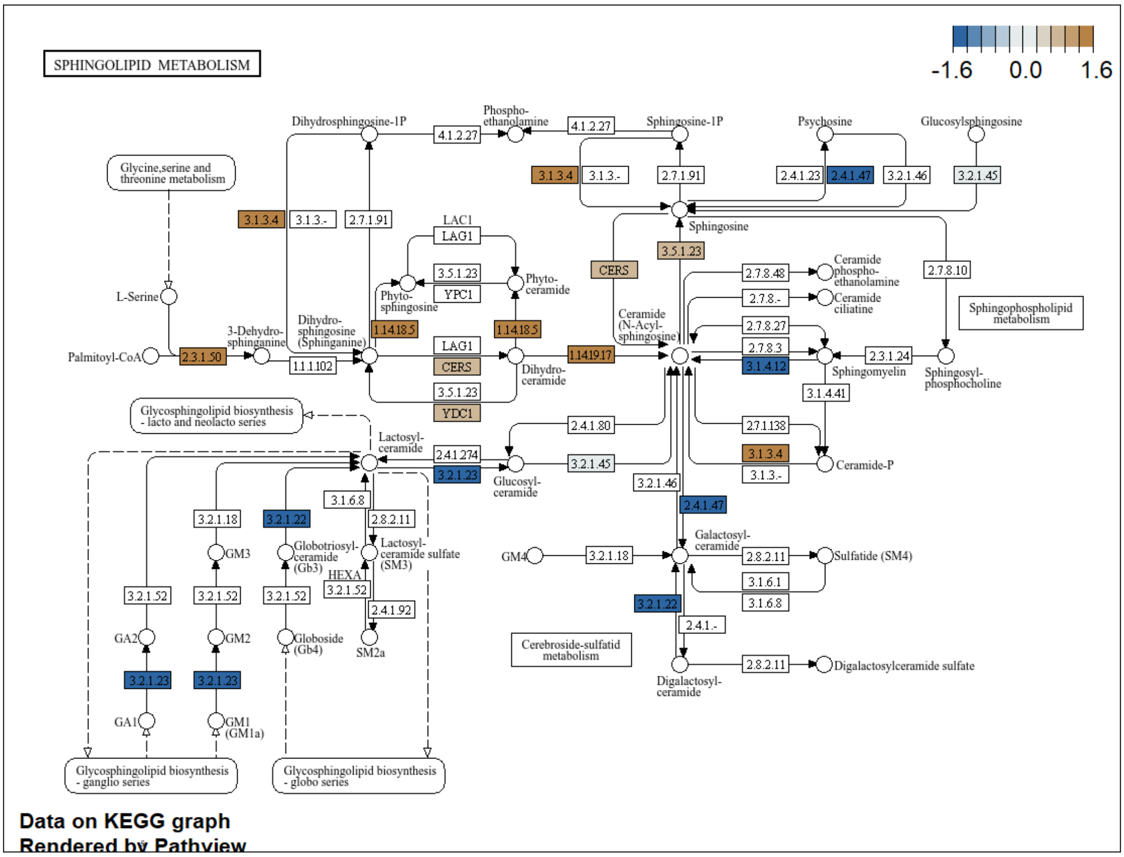

Figure S6. Pathway diagram of sphingolipid metabolism.

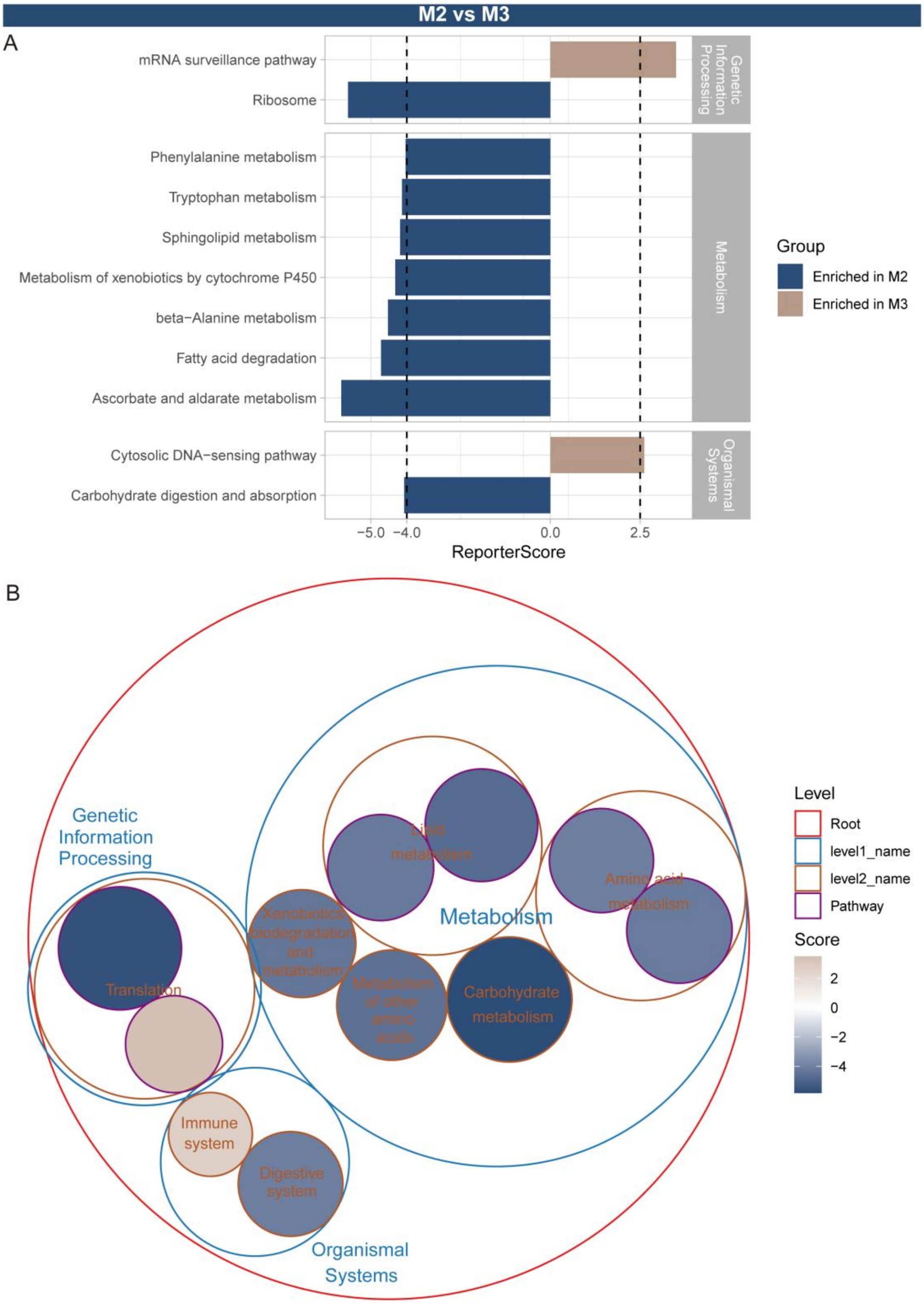

Figure S7. Comparative analysis of M2 and M3 tissues by GRSA.
